# Rebalancing Nutrients, Reinforcing Antioxidant and Osmoregulatory Capacity, and Improving Yield Quality in Drought-Stressed *Phaseolus vulgaris* by Foliar Application of a Bee-Honey Solution

**DOI:** 10.3390/plants12010063

**Published:** 2022-12-22

**Authors:** Sameera A. Alghamdi, Hesham F. Alharby, Atif A. Bamagoos, Safi-naz S. Zaki, Abdelmonam M. A. Abu El-Hassan, El-Sayed M. Desoky, Ibrahim A. A. Mohamed, Mostafa M. Rady

**Affiliations:** 1 Department of Biological Sciences, Faculty of Science, King Abdulaziz University, Jeddah 21589, Saudi Arabia; 2 Plant Biology Research Group, Department of Biological Sciences, Faculty of Science, King Abdulaziz University, Jeddah 21589, Saudi Arabia; 3 Department of Water Relations and Field Irrigation, National Research Centre, Dokki, Cairo 12622, Egypt; 4 Department of Food Science and Technology, Faculty of Agriculture, Fayoum University, Fayoum 63514, Egypt; 5 Botany Department, Faculty of Agriculture, Zagazig University, Zagazig 44511, Egypt; 6 Botany Department, Faculty of Agriculture, Fayoum University, Fayoum 63514, Egypt

**Keywords:** common bean, water limitation, growth and productivity, yield quality, antioxidant defense system, biostimulants

## Abstract

Bee-honey solution (BHS) is considered a plant growth multi-biostimulator because it is rich in osmoprotectants, antioxidants, vitamins, and mineral nutrients that can promote drought stress (DtS) resistance in common bean plants. As a novel strategy, BHS has been used in a few studies, which shows that the application of BHS can overcome the stress effects on plant productivity and can contribute significantly to bridging the gap between agricultural production and the steady increase in population under climate changes. Under sufficient watering (SW (100% of crop evapotranspiration; ETc) and DtS (60% of ETc)), the enhancing impacts of foliar application with BHS (0%, 0.5%, 1.0%, and 1.5%) on growth, productivity, yield quality, physiological-biochemical indices, antioxidative defense ingredients, and nutrient status were examined in common bean plants (cultivar Bronco). DtS considerably decreased growth and yield traits, green pod quality, and water use efficiency (WUE); however, application of BHS at all concentrations significantly increased all of these parameters under normal or DtS conditions. Membrane stability index, relative water content, nutrient contents, SPAD (chlorophyll content), and PSII efficiency (Fv/Fm, photochemical activity, and performance index) were markedly reduced under DtS; however, they increased significantly under normal or DtS conditions by foliar spraying of BHS at all concentrations. The negative impacts of DtS were due to increased oxidants [hydrogen peroxide (H_2_O_2_) and superoxide (O_2_^•−^)], electrolyte leakage (EL), and malondialdehyde (MDA). As a result, the activity of the antioxidant system (ascorbate peroxidase, glutathione reductase, catalase, superoxide dismutase, α-tocopherol, glutathione, and ascorbate) and levels of osmoprotectants (soluble protein, soluble sugars, glycine betaine, and proline) were increased. However, all BHS concentrations further increased osmoprotectant and antioxidant capacity, along with decreased MDA and EL under DtS. What is interesting in this study was that a BHS concentration of 1.0% gave the best results under SW, while a BHS concentration of 1.5% gave the best results under DtS. Therefore, a BHS concentration of 1.5% could be a viable strategy to mitigate the DtS impairment in common beans to achieve satisfactory growth, productivity, and green pod quality under DtS.

## 1. Introduction

Global warming, lack of precipitation, scarcity of fresh irrigation water (drought stress; DtS), environmental impairment, and increased salinization of water and soil began to be evident since the beginning of the twenty-first century [1]. Among the many issues that need to be addressed through sustainable agriculture is trying to overcome the problem of DtS. Many studies have addressed this problem and found many solutions [2,3,4,5], but it is necessary to find more effective solutions at the lowest possible costs. DtS is a destructive stress that limits crop production [6,7], given that the continuing decline in fresh irrigation water occurs in parallel with increased food production demand. This problem needs the raising of water use efficiency (WUE) [8]. DtS hinders plant growth and yield by inducing cell water loss, which impairs mitotic division and obstructs plant cell expansibility [9,10]. 

Under DtS, ABA (abscisic acid) accumulates to trigger several responses in plant cells [8,11]. As a secondary response, DtS stimulates the overproduction of ROS (reactive oxygen species) in plant organelles like peroxisome, mitochondria, and chloroplasts [12]. The excessive production of superoxide (O_2_^•−^) and hydrogen peroxide (H_2_O_2_), as the most important ROS, obstructs the normal balance between their production and removal [8]. ROS catalyze oxidative damage to proteins as highly essential cellular components. ROS affect cell function and disturb redox balance [9,12], besides causing degradation of chlorophylls, rupture of cell membranes, and decreasing membrane stability [8,12,13]. Cell death due to prolonged DtS may occur due to excessive ROS production that limits the scavenging action of the antioxidant machinery [14,15,16]. In order for plants to defend themselves against oxidative damage, they develop several adaptive mechanisms, which include upregulation of antioxidant defense system (ADS) activity. This ADS includes ROS-scavenging enzymes (including glutathione reductase (GR), catalase (CAT), superoxide dismutase (SOD), and ascorbate peroxidase (APX)) along with α-tocopherol, glutathione, and ascorbate as low-molecular-mass antioxidants [7,8,17]. Additionally, osmoprotectants (e.g., soluble sugars, proline, glycine betaine, etc.) accumulate for osmotic modulation and contribute to cell turgor maintenance [8,18]. Thus, under DtS, it is imperative to offer one or more sustainable strategies to uphold the plant to resist the effects of DtS. 

Biostimulants (BSts) are promising explorations as sustainable agricultural strategies to induce plant growth and production and uphold plants to overcome stress [4,5,19,20,21]. Applying the commercially available stimulants, including antioxidants and/or osmoprotective compounds, decreases the adverse impacts of various abiotic stresses, but they are very expensive for producers/farmers. However, natural BSts are inexpensive products or byproducts of plants or organisms. They have been actively contributing to sustainable agriculture rather than synthetic stimulants [8,20,22]. The foremost mechanisms that biostimulators target are closely related to the biostimulator nature. The chemical makeup of the biostimulator is complex and two or more compounds can act concurrently, so the complete characterization of the mode of action is not yet accurate [8,19,20,21] and needs further study. As one of the BSts, bee-honey solution (BHS) has been shown to be highly efficient in increasing the resistance of stressed plants to undesirable stress-induced effects [8,20]. The key direct-improving mechanisms of BHS are attributed to the fact that it contains many plant growth-promoting compounds, including osmoprotectants, antioxidants, vitamins, organic and inorganic acids, mineral nutrients, phenolic acids, and flavonoids, all of which can penetrate leaf cells after spraying the solution. In addition, BHS possesses a high DPPH radical-scavenging activity. All of these properties of BHS have been shown to induce physio-biochemical and antioxidant modifications, raise nutrient and water absorption, suppress ROS levels, and enhance resistance to stress—including DtS [8,20,23,24]. Further, flavonoids and enzymes in BHS prevent auto-oxidation [23] and are involved in the removal of ROS [24], providing functional protection from oxidative damage caused by DtS. In a field trial, BHS, as one the highly effective BSts, overcame DtS in faba bean plants [8]. Indeed, faba bean plants foliar sprayed with BHS showed higher dry biomass production, green pod yield, and WUE. Additionally, at the same time that BHS effectively suppressed oxidative stress biomarkers and their damage [O_2_^•−^, H_2_O_2_, malondialdehyde (MDA), and electrolyte leakage (EL)], it markedly increased the efficiency of photosynthetic machinery and different antioxidants, cell membrane integrity, leaf tissue water integrity, osmoprotectant content, and nutrient content.

In economic terms, *Phaseolus vulgaris* L. (‘common bean’ is its English name) is an important food legume in many of the world’s regions (Mexico, USA, Brazil, and many African countries), including Egypt [1]. In addition, common beans contribute to about 50% of the legumes consumed worldwide. Its seeds provide carbohydrates, vitamins, several mineral nutrients, and protein of high quality in human diets [25]. In order to develop and produce a seed yield, based on their genetic potential, common bean cultivars require large amounts of fresh irrigation water during the growing season [26]. Therefore, exposure to DtS can lead to diminished common bean growth, yield, and yield quality. The severity of decreasing growth and yield depends on time of exposure, severity of DtS, and stage of development. In arid and semi-arid regions, including Egypt, annual common bean yield losses can exceed 60–80% depending on the severity and duration of DtS [26,27].

Very few studies have so far been applied using BHS as natural BSts alone, or in combination with others, under DtS. Accordingly, this investigation aimed to evaluate the promoting impacts of BHS as a promising strategy to attenuate the negative impacts of DtS on common bean plants. The hypothesis confirmed in the current investigation was that two foliar applications of BHS would increase common bean growth and yield traits under DtS. This hypothesis was confirmed at the field level by using BHS at rates of 0.5, 1.0, and 1.5% as foliar sprays. Growth, yield, and green pod quality traits, efficiency of photosynthetic machinery, cell membrane integrity, leaf tissue integrity, contents of osmoprotectant, activity of different antioxidants, nutrient contents, and oxidative stress markers and their consequences (O_2_^•−^, H_2_O_2_, MDA, and EL) were checked.

## 2. Materials and Methods

### 2.1. The Location and Analysis of the Soil Used

A 600 m^2^ piece of loamy sand texture soil on a private farm (29.3452 N, 30.5686 E) located in Fayoum, Egypt, was allocated for two field trials in the summer seasons 2021 and 2022. According to [28], hypothermic, siliceous, and typical Torripsamments are the tested soil classifications. The soil’s physicochemical properties (Table 1) were performed [29,30]. Classifications listed in [31] indicated that the soil is non-saline with an ECe of 2.54 dS m^−1^.

### 2.2. Seed Sowing, Trial Layout and Treatments

The Egyptian Center for Agricultural Research was the source of the *P. vulgaris* seeds (cultivar Bronco). Sodium hypochlorite was used to prepare a 1% (*v*/*v*) solution to sterilize the seeds for 2 min, and then distilled water was utilized to clean the seeds from the sterilizing solution. After drying the seeds at room temperature, they were planted on February 25 in the 2021 and 2022 seasons, and the crop duration was 75 days. After full emergence, seedlings were reduced to two per hill. The experimental soil piece was divided into plots of 10.8 m^2^ each [3 m (6 rows) × 3.6 m], and the distances were 60 cm between rows and 20 cm between hills (the plant densities are 180 plants plot^−1^ and about 162,000 plants ha^−1^).

Both the 2021 and 2022 experiments were performed by arranging the treatments in a split-plot in a randomized complete block layout. Each treatment was replicated in six plots. There are two experimental factors for this study. The first is irrigation regimes (100% and 60% of the crop evapotranspiration; ETc) that were assigned to main plots. The second is the bee-honey solution (BHS) foliar applications that were assigned to sub-plots. The BHS was applied at 0 (control), 0.5, 1.0, and 1.5% as a foliar spray. Both irrigation regimes (sufficient watering (SW) and drought stress (DtS)) were separated by 2 m of unirrigated area. Until the seedlings were well established (two weeks after sowing; WAS), common bean plants were watered well (100% ETc). Then, the two irrigation treatments (SW and DtS) were launched. As the irrigation treatments commenced, foliar spraying with different concentrations of BHS was applied. Two and four weeks after the first spray, the second and third sprays were performed. Different BHSs were sprayed at 1.2, 1.5, and 1.8 L plot^−1^ for the three sprays, respectively. The spray solutions were provided with a 0.1% (*v*/*v*) solution of Tween-20 as a surfactant for optimum penetration into the leafy tissue. Following the Egyptian Center for Agricultural Research recommendations, different NPK fertilizers (e.g., 300 kg NH_4_NO_3_ (33% N) per hectare, 200 kg calcium superphosphate (12% P_2_O_5_) per hectare, and 200 kg potassium sulfate (50% K_2_O) per hectare) and other agricultural practices were utilized. The physicochemical composition of fresh raw clover honey is presented in Table 2.

### 2.3. Irrigation Water Applied; IWA

Reference evapotranspiration (ETo) was set utilizing the data of class A pan (E_pan_ (mm per day)), neighboring plots adjusted for a fitting pan coefficient (K_pan_) and crop coefficient (K_c_) [32]. The ETc (in mm day^−1^) was assessed by applying Allen’s equation [32]: ETc = E_pan_ × K_pan_ × K_c_

IWA (m^3^) was computed utilizing the following equation:

IWA = [(A × ETc × Ii × Kr)/(Ea × 1000 × (1 − LR))]

where A is area of plot (m^2^), ETc is the water requirements of the used crop (mm day^−1^), Ii are the intervals of irrigations (day), Kr is the covering factor, Ea is the efficiency of application (%), and LR are the leaching requirements. 

The total IWA during the 2021 and 2022 seasons was 2620 and 2612 m^3^ ha^−1^ for 100% of ETc, and 1572 and 1567 m^3^ ha^−1^ for 0% of ETc. HH2 digital hygrometer sensors (Cambridge, UK) were used for the content of water of the soil at 2-day intervals at a depth of 0–30 cm.

### 2.4. Growth and Yield Characteristics, WUE, and Pod Quality Traits

In each season, plant growth traits were analyzed in ten seven-week-old plants randomly chosen from each sub-main plot. For each plant, the number of leaves was recorded and the area of leaves (cm^2^) was taken utilizing a Planix 7 held-hand planimeter (Tamaya Technics Inc., Tokyo, Japan). Shoots were subjected to oven drying (at 70 ± 2 °C) for recording dry weight once constant weights were reached. In the marketable green pod stage (starting at 9 WAS), 5 harvests were made at 3-day intervals. The number of green pods plant^−1^ and pod yield (ton ha^−1^) were measured utilizing four rows from each experimental plot, excluding the two rows assigned to the parameters of growth, physio-biochemistry, and antioxidant defense system components. The WUE was computed using the equation of Jensen [33]:

WUE = Green pod yield (kg per ha)/IWA (kg pods per m^3^)

The content of pod protein (%) was determined in the dried pod samples using the micro-Kjeldahl apparatus (Ningbo Medical Instruments Co., Ningbo, China) (pod protein (%) = total N (%) × 6.25) [34]. Total pod carbohydrate content (%) was estimated in dried pod samples by applying the method of anthrone [35]. Fiber (%) in pods was estimated by applying the procedures in [36].

### 2.5. Assessment of the Photosynthetic Machinery Efficiency

A SPAD-502 Chlorophyll Meter (Minolta Sensing, Inc., Osaka, Japan) was utilized to assess the greenness of plant leaf (SPAD). In fresh leaves, photochemical activity was estimated using the technique of Ferricyanide [37]. Fluorescence of chlorophyll—a was assessed utilizing a PEA Chlorophyll Fluorometer (Hansatech Instruments Ltd., Kings Lynn, UK). PSII maximum quantum yield (F_v_/F_m_) was determined by applying the equation: F_v_/F_m_ = (F_m_ − F_0_)/F_m_ [38]. According to [39], photosynthesis performance index (PI_ABS_) was computed as follows:

PI_ABS_ = (1 − F0/Fm)/(M0/Vj) × (Fm − F0)/F0) × (1 − Vj/Vj)

where F0 is fluorescence intensity at 50 μs, Fm is maximum fluorescence intensity, M0 is the initial slope of fluorescence kinetics which is derived from the equation: M0 = 4 (F300 μs − F0)/(Fm − F0), Vj is relative variable fluorescence at 2 ms calculated as Vj = (Fj − F0)/(Fm − F0), and Fj is fluorescence intensity at the j step (at 2 ms).

### 2.6. Assessment of Nutrient Contents

Dried leafy and pod samples were digested in an acidic mixture (1 volume perchloric acid + 3 volumes of nitric acid). The digestion solution was applied to estimate K^+^, P, and N contents (mg g^−1^ DW). The methods in A.O.A.C. [34] were applied to estimate N content utilizing the micro-Kjeldahl (Medical Instruments Co., Ningbo, China) method. The method of Jackson [40] was applied to estimate P content (mg g^−1^ DW). The method is based on the rate of reduction of molybdo-phosphoric in H_2_SO_4_ by molybdenum. The method of Page et al. [29] was applied to estimate K^+^ content (mg g^−1^ DW) utilizing a Perkin-Elmer Model 52-A Flame Photometer (Glenbrook, Stamford, CT, USA). The procedures in [41] were applied to estimate leaf contents of Zn, Mn, and Fe (µg g^−1^ DW), utilizing Atomic Absorption with samples of standard reference (NIST, Gaithersburg, MD, USA).

### 2.7. Relative Water Content and Membrane Stability Index

The method of Osman and Rady [42] was practiced to estimate relative water content (RWC) in leaf tissue. The method is based on measuring the fresh weight (Fwt), turgid weight (Twt), and dry weight (Dwt) of a number of leaf blade discs, and the RWC was calculated as:

RWC (%) = [(Fwt − Dwt)/(Twt − Dwt)] × 100

The method of Rady [43] was applied to estimate the membrane stability index (MSI). The method is based on measuring the electrical conductivity (EC) of a solution containing leaf tissue after heating to 40 °C (EC_I_) and boiling at 100 °C (EC_II_), and the MSI was calculated as:

MSI (%) = [1 − (EC_I_/EC_II_)] × 100

### 2.8. Markers of Oxidative Stress and Their Consequences

The method of Madhava Rao and Sresty [44] was applied to estimate the peroxidation of lipids through estimating malondialdehyde (MDA) content (A_532–600_ g^−1^ FW), utilizing “155 mM^−1^ cm^−1^” as an extinction coefficient. The method of Velikova et al. [45] was applied to estimate superoxide (O_2_^•−^) content (µmol g^−1^ FW). The method is based on immersing leaf fragments for 1 h in a buffer solution consisting of 10 mM K-phosphate (pH 7.8) + 10 mM NaN_3_ + 0.05% NBT. The O_2_^•−^ content was calculated from readings taken at 580 nm. The method of Kubiś [46] was applied to colorimetrically quantify hydrogen peroxide (H_2_O_2_ in µmol g^−1^ FW) at 390 nm using appropriate standard curves.

The method of Rady and Rehman [47] was applied to estimate the leakage of electrolytes (EL) in leaf tissue. The method is based on measuring the EC of a solution containing leaf tissue in normal (25 °C) (EC_0_), heated (45–55 °C) (EC_I_), and boiled solution (100 °C) (EC_II_), and the MSI was calculated as follows:

EL (%) = [(EC_I_ − EC_0_)/EC_II_] × 100

### 2.9. Assessment of Osmoprotectants and Non-Enzymatic Antioxidant Compound Contents

The method of Irigoyen et al. [48] was applied to assess total leaf soluble sugars content (mg g^−1^ DW). The method is based on obtaining an ethanol extract that reacted with a freshly prepared anthrone reagent in 72% H_2_SO_4_, and after boiling for 10 min and then cooling, 625 nm was adjusted to record the absorbance. The method of Bates et al. [49] was applied to estimate proline content (μM g^−1^ DW) based on the use of toluene to obtain an extract and 520 nm was adjusted to record the absorbance, and appropriate standard curves were used. The method of Grieve and Grattan [50] was utilized to evaluate leaf glycine betaine (GB) content (μM g^−1^ DW). The method is based on the reaction of GB extract with cold KI–I_2_ as a reagent, and the periodide crystals formed were read at 365 nm. In leaf tissue homogenates, ascorbate and glutathione contents (in µM g^−1^ FW) were estimated by applying the full procedures in [51,52]. Additionally, α-tocopherol content (in µM g^−1^ DW) was estimated following the procedures in [53].

### 2.10. Activity Assays of Antioxidant Enzymes

Leafy enzymatic extract was prepared from 500 mg using an ice-cold buffer of potassium-phosphate, pH 7.0, containing PVP (1%). Under 4 °C, the homogenates were subjected to 15-min centrifugation (12,000× *g*) to obtain the enzymatic extract, used to assay activities of enzymes, except for SOD. All enzyme activities were measured in Unit mg^−1^ protein. The method of Aebi [54] was practiced to measure CAT activity, and 240 nm was utilized to record the absorbances. The method is based on the decomposition rate of the H_2_O_2_ for 2 min by the enzyme in the enzymatic extract. The method of Nakano and Asada [55] was practiced to assay APX activity. The method is based on the decomposition rate of the H_2_O_2_ for 2 min by the enzyme in the enzymatic extract in the presence of EDTA and ascorbate. The method of Foster and Hess [56] was practiced to measure GR activity. The method is based on the reduction rate of GSSG (oxidized glutathione) for 3 min by the enzyme in the enzymatic extract in the presence of EDTA and NADPH. To assay SOD and soluble protein, a frozen sample was homogenized with ice, and the homogenate was centrifuged to obtain a functional extract. Overnight, the extract was dialyzed to uproot the interference in an SOD assay from low-molecular mass substances. The method of Yu and Rengel [57] was practiced to measure SOD activity. The method is based on the inhibition rate of NBT photochemical reduction. The method of Bradford [58] was applied to estimate the total leaf content of soluble protein (mg g^−1^ DW).

### 2.11. Statistical Tests

The treatments were ordered in a split-plot design and the data were exposed to two-way ANOVA. The homogeneity of error variance was tested before beginning of the analyses [59], as well as for normality distribution [60]. Among means, significant differences were assessed at 1% and 5% levels of probability (*p* ≤ 0.01 and 0.05) by applying Tukey’s HSD (honestly significant difference) test applying the GenStat 17th Ed. (VSN International Ltd., Hemel Hempstead, UK) software.

## 3. Results

To explore the potential enhancing impacts of a bee-honey solution (BHS) foliarly sprayed at three concentrations (0.5% (BHS-1), 1.0% (BHS-2), and 1.5% (BHS-3)) against distilled water (0% (BHS-0) as a control) on *P. vulgaris* plants under normal conditions (NmC) and drought stress (DtS), thirty-three traits were estimated and presented below. An interesting finding was that BHS-2 achieved the best results under NmC, while the best results reported under DtS were with BHS-3.

Table 2 shows the analysis of fresh raw clover honey. It has a pH of 4.02 and is rich in proteins, organic acids, and osmoprotective compounds (e.g., soluble sugars and amino acids, including proline). It is also rich in essential nutrients (e.g., Se, I, Cu, Zn, Mn, Fe, S, Ca, Mg, P, and K), as well as antioxidants and vitamin C (ascorbate) and B-group vitamins (e.g., B1, B2, B3, B5, B6, and B9). Furthermore, it possesses a potent activity of DPPH radical scavenging (89.4%). These essential bioactive components make this type of bee-honey a valuable multi-biostimulator. Therefore, the use of BHS at precise concentration (e.g., 1.0−1.5%) is a useful strategy to rebalance mineral nutrients and reinforce the capacity of osmoprotectants and antioxidant compounds as defense mechanisms in *P. vulgaris* plants under DtS conditions.

### 3.1. Growth and Yield Traits, and Water-Use Efficiency (WUE)

**Figure 1:**
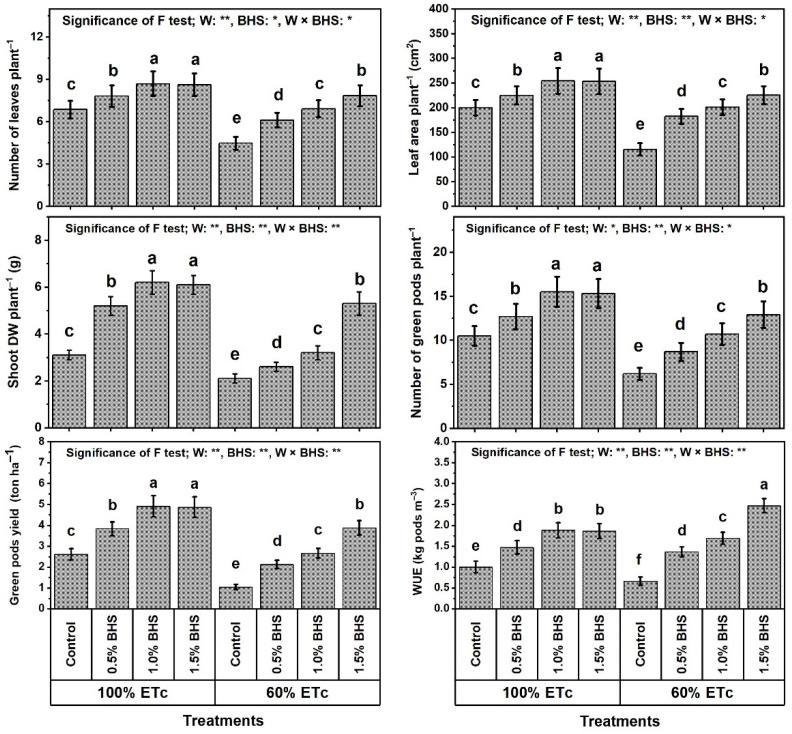
Foliar application influences of bee-honey solution (BHS) on growth and green pod yield components of *Phaseolus vulgaris* plants grown under sufficient watering (100% of crop evapotranspiration; ETc) or drought stress (60% of ETc). DW: dry weight, and WUE: water use efficiency. * and ** indicate differences at *p* ≤ 0.05 and *p* ≤ 0.01 probability levels, respectively. Columns labeled by the same letter in each plot are not significantly different according to the LSD test (*p* ≤ 0.05).

The findings in Figure 1 show that the growth traits and green pod yield of *P. vulgaris* plants such as plant leaf number (NLP), plant leaf area (LAP), shoot dry weight per plant (SDWP), number of green pods per plant (NGPP), green pod yield per hectare (GPYH), and WUE were significantly decreased under DtS by 34.8, 42.2, 32.3, 41.0, 60.2, and 34.0%, respectively.

However, foliar spray with different BHS concentrations (e.g., 0.5−1.5%) noticeably increased all of these traits under NmC and DtS compared to the corresponding controls. Under NmC, all BHS concentrations significantly increased NLP, LAP, SDWP, NGPP, GPYH, and WUE. The best concentration that yielded the best results was BHS-2, which increased these traits by 26.7, 27.2, 100.0, 47.6, 88.1, and 88.0%, respectively, compared to the corresponding control. Under DtS, all BHS concentrations significantly increased NLP, LAP, SDWP, NGPP, GPYH, and WUE. The best concentration that yielded the best results was BHS-3, which increased these traits by 75.4, 95.1, 152.4, 108.1, 273.1, and 274.2%, respectively, in comparison to the corresponding control. The increases induced by BHS were more pronounced under DtS than under NmC. Moreover, the results obtained with BHS-3 under DtS exceeded those of the unstressed control by 14.3, 12.8, 71.0, 22.9, 48.7, and 147% for NLP, LAP, SDWP, NGPP, GPYH, and WUE, respectively.

### 3.2. Pod Quality Parameters

**Figure 2:**
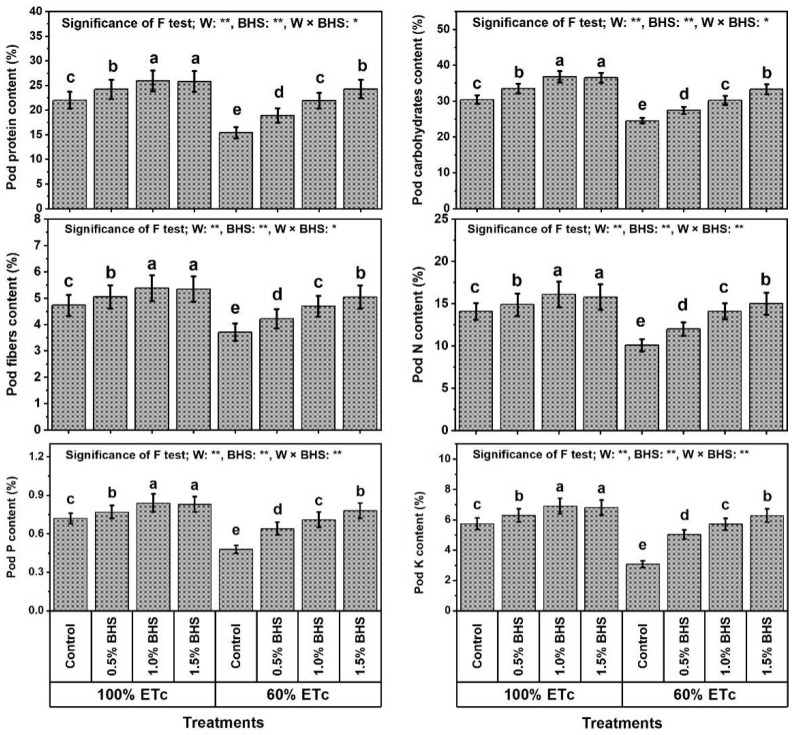
Foliar application influences of bee-honey solution (BHS) on green pod quality of *Phaseolus vulgaris* plants grown under sufficient watering (100% of crop evapotranspiration; ETc) or drought stress (60% of ETc). N: nitrogen, P: phosphor, K: potassium. * and ** indicate differences at *p* ≤ 0.05 and *p* ≤ 0.01 probability levels, respectively. Columns labeled by the same letter in each plot are not significantly different according to the LSD test (*p* ≤ 0.05).

The findings in Figure 2 show that the common bean pod protein, carbohydrate, fiber, N, P, and K contents were significantly decreased under DtS by 30.0, 19.4, 21.6, 28.4, 33.3, and 46.3%, respectively.

However, foliar spray with different BHS concentrations (e.g., 0.5−1.5%) noticeably increased all of these traits under NmC and DtS compared to the corresponding controls. Under NmC, all BHS concentrations significantly increased pod protein, carbohydrate, fiber, N, P, and K contents. The best concentration that yielded the best results was BHS-2, which increased these traits by 17.7, 21.1, 13.7, 14.2, 16.7, and 20.4%, respectively, compared to the corresponding control. Under DtS, all BHS concentrations significantly increased pod protein, carbohydrate, fiber, N, P, and K contents. The best concentration that yielded the best results was BHS-3, which increased these traits by 57.8, 35.9, 35.8, 48.5, 62.5, and 103.9%, respectively, in comparison with the corresponding control. The increased contents of pod protein, carbohydrates, fibers, K, P, and N by BHS were more pronounced under DtS than under NmC. Moreover, the results obtained with BHS-3 under DtS exceeded those of the unstressed control by 10.5, 9.5, 6.6, 6.4, 8.3, and 9.4% for pod protein, carbohydrate, fiber, N, P, and K contents, respectively.

### 3.3. Photosynthetic Efficiency, Mineral Nutrient and Protein Contents, and Leaf Integrity

**Figure 3:**
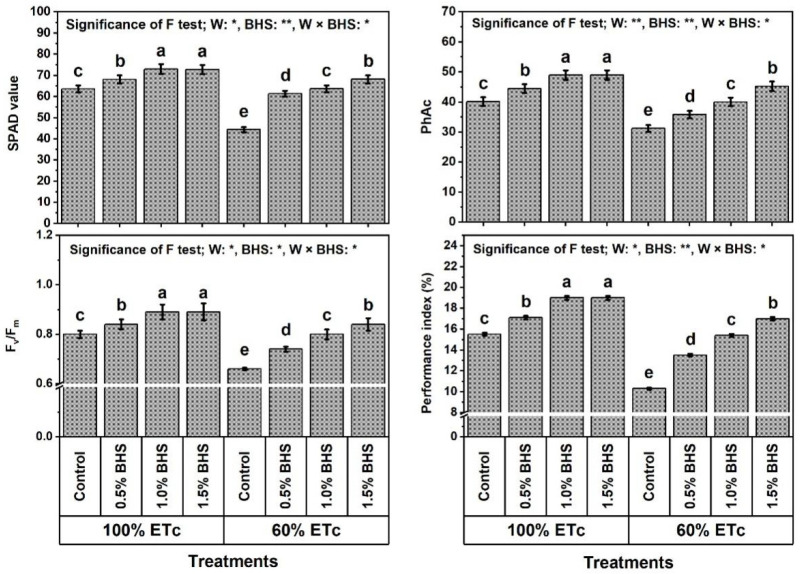
Foliar application influences of bee-honey solution (BHS) on photosynthetic machinery efficiency of *Phaseolus vulgaris* plants grown under sufficient watering (100% of crop evapotranspiration; ETc) or drought stress (60% of ETc). SPAD: Soil Plant Analysis Development (an indicator of chlorophyll content), PhAc: photochemical activity, and Fv/Fm: efficiency of PSII. * and ** indicate differences at *p* ≤ 0.05 and *p* ≤ 0.01 probability levels, respectively. Means followed by the same letter in each column are not significantly different according to the LSD test (*p* ≤ 0.05).

**Figure 4:**
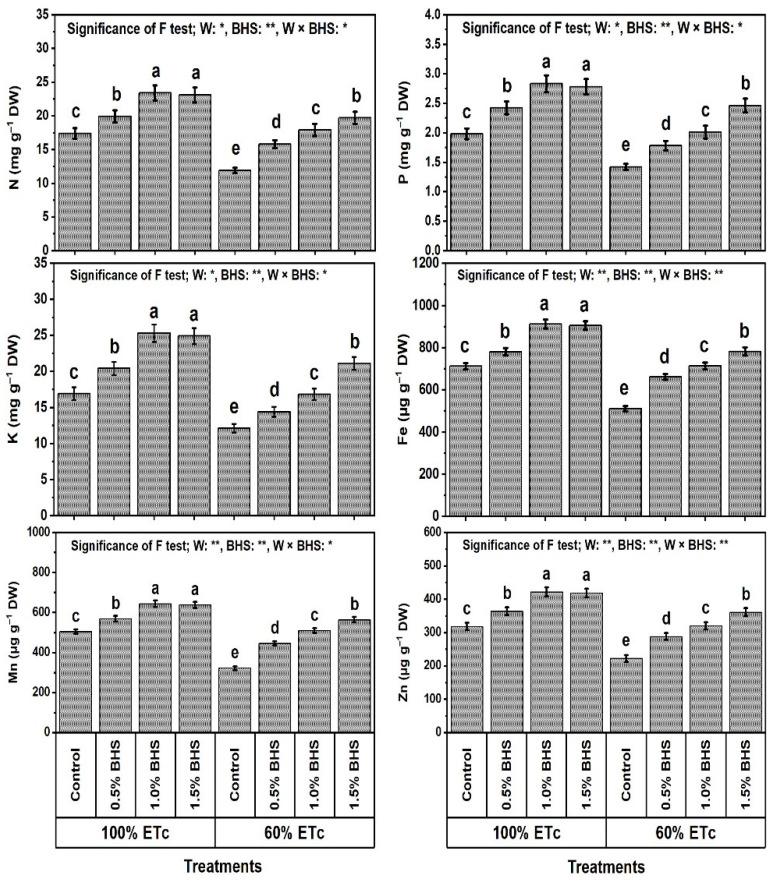
Foliar application influences of bee-honey solution (BHS) on the contents of mineral nutrients of *Phaseolus vulgaris* plants grown under sufficient watering (100% of crop evapotranspiration; ETc) or drought stress (60% of ETc). DW: dry weight, N: nitrogen, P: phosphorus, K: potassium, Fe: iron, Mn: manganese, and Zn: zinc. * and ** indicate differences at *p* ≤ 0.05 and *p* ≤ 0.01 probability levels, respectively. Columns labeled by the same letter in each plot are not significantly different according to the LSD test (*p* ≤ 0.05).

The findings in Figure 3 and Figure 4 show that DtS significantly decreased the photosynthetic efficiency indices (e.g., SPAD value by 30.2%, photochemical activity (PhAc) by 22.2%, Fv/Fm by 17.5%, and performance index (PI) 33.5%) and mineral nutrient contents (N by 31.6%, P by 28.3%, K by 28.4%, Fe by 28.2%, Mn by 36.1%, and Zn by 30.2%).

**Figure 5:**
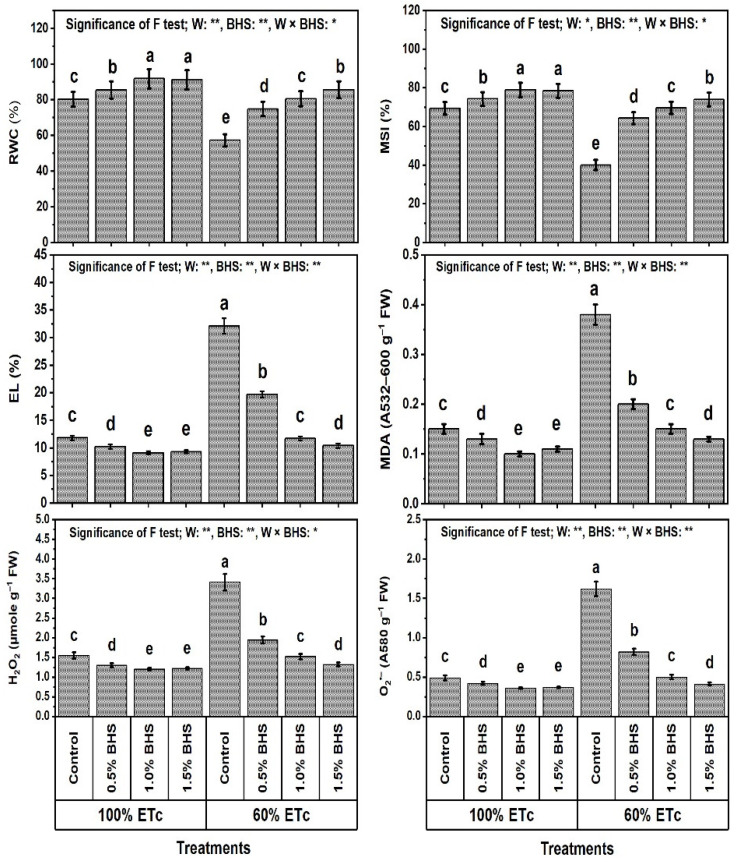
Foliar application influences of bee-honey solution (BHS) on the health of leafy tissues and the levels of oxidative stress markers in *Phaseolus vulgaris* plants grown under sufficient watering (100% of crop evapotranspiration; ETc) or drought stress (60% of ETc). FW: fresh weight, RWC: relative water content, MSI: membrane stability index, EL: electrolyte leakage, MDA: malondialdehyde (an indicator of lipid peroxidation), H_2_O_2_: hydrogen peroxide, and O_2_^•−^: superoxide radical. * and ** indicate differences at *p* ≤ 0.05 and *p* ≤ 0.01 probability levels, respectively. Columns labeled with the same letter in each column are not significantly different according to the LSD test (*p* ≤ 0.05).

It also decreased relative water content (RWC) by 28.7% and membrane stability index (MSI) by 42.2% (Figure 5). However, leafy treatments with different concentrations of BHS (e.g., 0.5, 1.0, and 1.5%) noticeably increased all of the above traits under NmC and DtS compared to the corresponding controls. Under NmC, all BHS concentrations significantly increased SPAD, PhAc, Fv/Fm, PI, RWC, MSI and total soluble protein, as well as N, P, K, Fe, Mn, Zn contents. The best concentration that conferred the best findings was BHS-2, increasing these traits by 14.8, 21.9, 11.3, 22.6, 14.5, 13.7, 21.0, 34.5, 42.9, 49.7, 28.1, 27.8, and 32.7%, respectively, in comparison to the corresponding control. Under DtS, all BHS concentrations significantly increased all of the above traits, and the best concentration that gave the best results was BHS-3. It markedly increased these traits by 53.7, 44.9, 27.3, 65.0, 65.5, 73.2, 74.4, 53.0, 75.2, 62.6, 49.7, 84.5, and 117.0%, respectively, compared with the corresponding control. The increases in all of the above traits by BHS were more pronounced under DtS than under NmC. Moreover, the results obtained with BHS-3 under DtS outperformed those of the unstressed control by 7.2, 12.7, 5.0, 9.7, 6.7, 6.6, 11.0, 13.2, 24.2, 24.9, 9.8, 11.9, and 13.5% for SPAD, PhAc, Fv/Fm, PI, RWC, MSI and total soluble protein, as well as N, P, K, Fe, Mn, Zn contents, respectively.

### 3.4. Relative Water Content, Membrane Stability Index, and Levels and Consequences of Markers of Oxidative Stress

The findings in Figure 5 show that DtS significantly decreased leaf relative water content (RWC) and membrane stability index (MSI) by 28.7 and 42.2%, respectively, while the levels of oxidants (H_2_O_2_; hydrogen peroxide, and O_2_^•−^; superoxide radical) and their damage in terms of EL (electrolyte leakage) and MDA (malondialdehyde as an indicator of lipid peroxidation) were noticeably elevated under DtS by 172.0, 153.3, 120.0, and 230.6%, respectively.

However, foliar spray with different BHS concentrations (e.g., 0.5−1.5%) noticeably increased RWC and MSI, while decreasing H_2_O_2_, O_2_^•−^, EL, and MDA under NmC and DtS compared to the corresponding controls. The best concentration that yielded the highest values of RWC and MSI and the lowest values of the oxidants and their consequences was BHS-2, which decreased H_2_O_2_ by 22.9%, O_2_^•−^ by 33.3%, EL by 22.6%, and MDA by 26.5% in comparison with the corresponding control. Under DtS, all BHS concentrations significantly increased RWC and MSI, and decreased H_2_O_2_, O_2_^•−^, EL, and MDA levels. The best concentration that yielded the lowest values was BHS-3, which decreased H_2_O_2_ by 67.6%, O_2_^•−^ by 65.8%, EL by 61.3%, and MDA by 74.7% in comparison with the corresponding control.

### 3.5. Osmoprotectant Contents, and Non-Enzymatic and Enzymatic Antioxidant Activities

**Figure 6:**
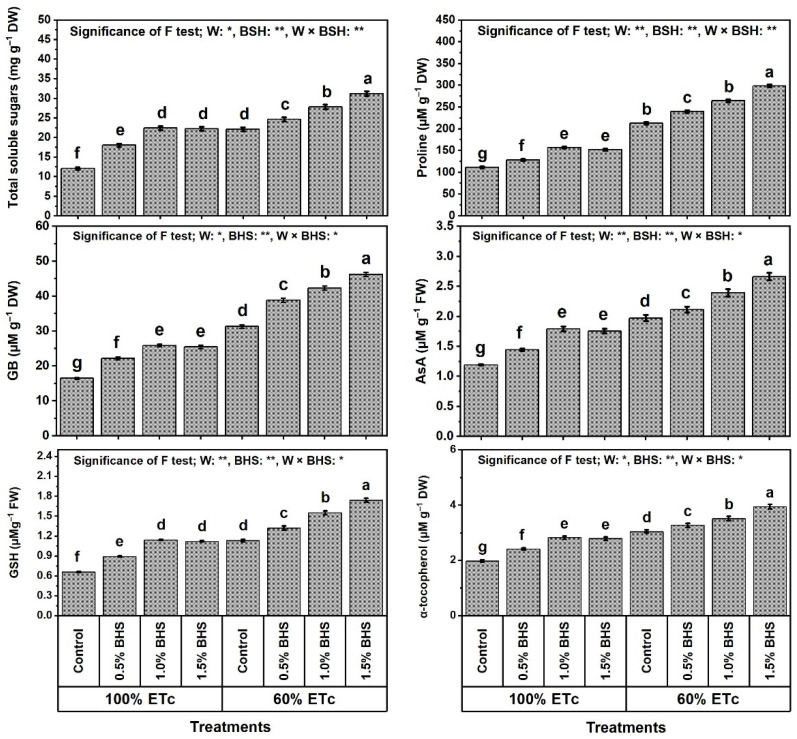
Foliar application influences of bee-honey solution (BHS) on the contents of osmoprotectant and non-enzymatic antioxidant compounds of *Phaseolus vulgaris* plants grown under sufficient watering (100% of crop evapotranspiration; ETc) or drought stress (60% of ETc). GB: glycine betaine, AsA: ascorbate, and GSH: glutathione. * and ** indicate differences at *p* ≤ 0.05 and *p* ≤ 0.01 probability levels, respectively. Columns labeled with the same letter in each plot are not significantly different according to the LSD test (*p* ≤ 0.05).

**Figure 7:**
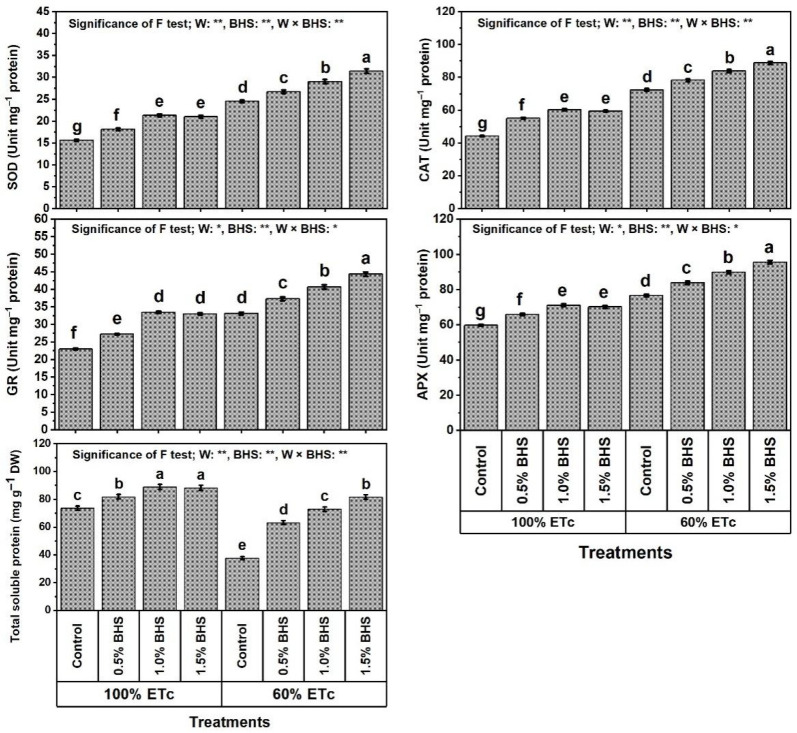
Foliar application influences of bee-honey solution (BHS) on the activities of antioxidant enzymes of *Phaseolus vulgaris* plants grown under sufficient watering (100% of crop evapotranspiration; ETc) or drought stress (60% of ETc). SOD: superoxide dismutase, CAT: catalase, GR: glutathione reductase, and APX: ascorbate peroxidase. * and ** indicate differences at *p* ≤ 0.05 and *p* ≤ 0.01 probability levels, respectively. Columns labeled with the same letter in each plot are not significantly different according to the LSD test (*p* ≤ 0.05).

The findings in Figure 6 and Figure 7 show that the levels of soluble sugars, proline, glycine betaine (GB), ascorbate (AsA), glutathione (GSH), α-tocopherol (αToc), and total soluble proteins, and SOD, CAT, GR, and APX activities in common bean plants were significantly increased under DtS by 82.6, 90.8, 90.9, 65.5, 71.2, 53.5, 48.8, 57.1, 63.6, 43.9, and 28.3%, respectively.

Moreover, foliar spray with different BHS concentrations (e.g., 0.5−1.5%) noticeably increased all of these traits under NmC and DtS compared to the corresponding controls. Under NmC, all BHS concentrations significantly increased the levels of soluble sugars, proline, GB, AsA, GSH, and αToc, and SOD, CAT, GR, and APX activities. The best concentration that yielded the highest values of these parameters was BHS-2, which increased them by 85.1, 40.8, 57.3, 50.4, 72.7, 42.4, 36.5, 36.2, 45.2, and 19.1%, respectively, in comparison to the corresponding control. Under DtS, all BHS concentrations further increased these parameters. The best concentration that yielded the highest values was BHS-3, which increased soluble sugars by 41.2%, proline by 40.4%, GB by 47.6%, AsA by 35.0%, GSH by 54.0%, αToc by 29.5%, SOD by 28.2%, CAT by 22.8%, GR by 33.8%, and APX by 24.8%, in comparison to the corresponding control.

### 3.6. Relationships 

**Figure 8:**
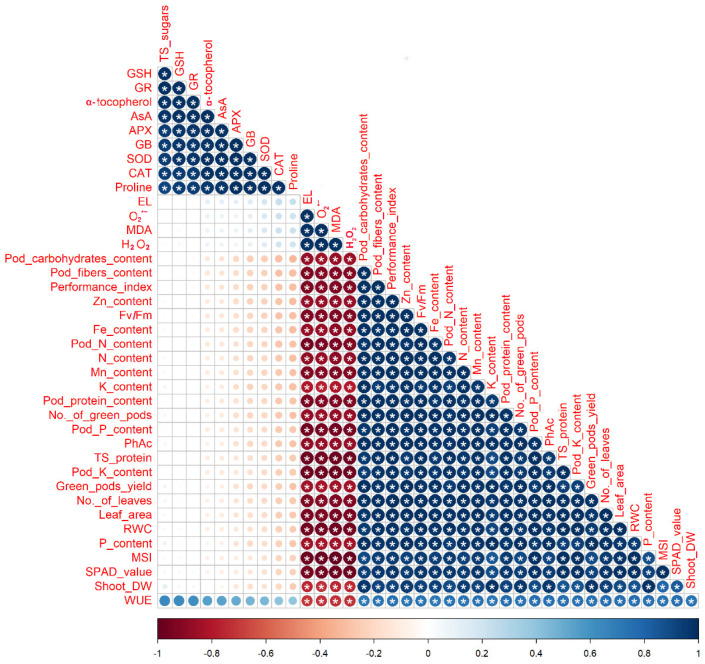
Graph of Pearson’s correlation analysis among the different studied parameters. The colors represent variations in the obtained data. SPAD: Soil Plant Analysis Development (an indicator of chlorophyll content), PhAc: photochemical activity, Fv/Fm: efficiency of PSII, DW: dry weight, N: nitrogen, P: phosphorus, K: potassium, Fe: iron, Mn: manganese, Zn: zinc, FW: fresh weight, RWC: relative water content, MSI: membrane stability index, EL: electrolyte leakage, MDA: malondialdehyde (an indicator of lipid peroxidation), H_2_O_2_: hydrogen peroxide, O_2_^•−^: superoxide radical, TS sugars: total soluble sugars, GB: glycine betaine, AsA: ascorbate, GSH: glutathione, SOD: superoxide dismutase, CAT: catalase, GR: glutathione reductase, APX: ascorbate peroxidase, WUE: water use efficiency, and TS protein: total soluble protein. * indicates the significant at *p* ≤ 0.05.

The relationship between the parameters in *P. vulgaris* plants grown under two irrigation regimes and fortified with BHS (Figure 8) was tested using Pearson’s correlation. 

**Figure 9:**
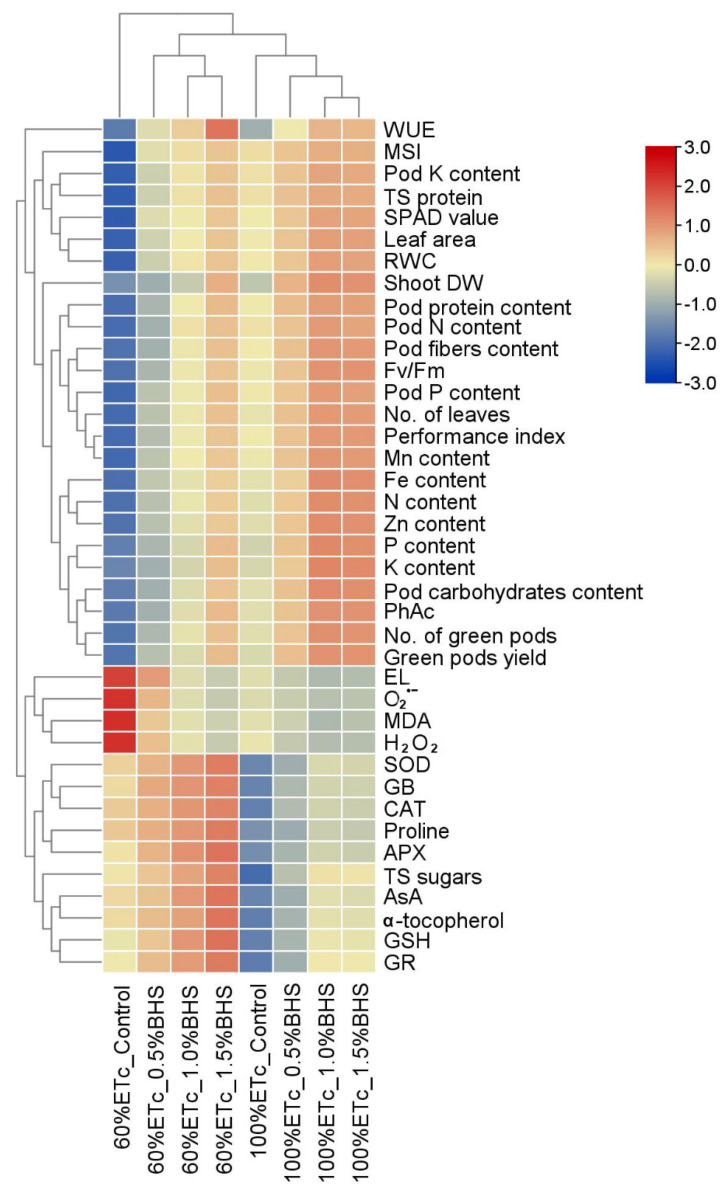
Heat map graph shows analysis of hierarchical clustering among the different studied parameters and treatments. The colors represent variations in the obtained data. SPAD: Soil Plant Analysis Development (an indicator of chlorophyll content), PhAc: photochemical activity, Fv/Fm: efficiency of PSII, DW: dry weight, N: nitrogen, P: phosphorus, K: potassium, Fe: iron, Mn: manganese, Zn: zinc, FW: fresh weight, RWC: relative water content, MSI: membrane stability index, EL: electrolyte leakage, MDA: malondialdehyde (an indicator of lipid peroxidation), H_2_O_2_: hydrogen peroxide, O_2_^•−^: superoxide radical, TS sugars: total soluble sugars, GB: glycine betaine, AsA: ascorbate, GSH: glutathione, SOD: superoxide dismutase, CAT: catalase, GR: glutathione reductase, APX: ascorbate peroxidase, WUE: water use efficiency, and TS protein: total soluble protein.

The obtained findings indicated a positive (significant, *p* ≤ 0.05) correlation between shoot DW, leaf area, number of leaves, green pods yield, number of green pods, and pod contents of protein, N, fibers, carbohydrates, and K with RWC, SPAD, total soluble protein, PhAc, Fv/Fm, performance index, and contents of Zn, Fe, N, Mn, K^+^, and P. Meanwhile, the above-mentioned parameters were negatively (significant, *p* ≤ 0.05) correlated with EL, O_2_^•−^, MDA, and H_2_O_2_ levels. Moreover, contents of total soluble sugars, α-tocopherol, GB, ASA, and CAT, SOD, APX, and GR activities showed a positive (significant, *p* ≤ 0.05) correlation with each other (Figure 8). In addition, to detect the interactive relation between the measured parameters and the treatments of foliar spray with BHS for *P. vulgaris* plants grown under DtS, a heatmap with hierarchical analysis was conducted (Figure 9). 

The hierarchical cluster analysis divided different treatments into three main groups. The 60% of ETc-control treatment alone was clustered in the first main group. The treatments of 60% of ETc-0.5% BHS, 60% of ETc-1.0% BHS, and 60% of ETc-1.5% BHS were clustered in the second main group that performed better than 60% of the ETc-control treatment. The treatments of 100% of ETc-control, 100% of ETc-0.5% BHS, 100% of ETc-1.0% BHS, and 100% of ETc-1.5% BHS were clustered in the third main group. The second main group was divided into two groups: 60% of ETc-0.5% BHS treatment that performed lower than 60% of ETc-1.0% BHS, and 60% of ETc-1.5% BHS treatments. The third main group was divided into two groups: 100% of ETc-control treatment that performed lower than 100% of ETc-0.5% BHS, 100% of ETc-1.0% BHS, and 100% of ETc-1.5% BHS treatments. These results indicated that foliarly sprayed BHS alleviated the negative impacts of DtS in *P. vulgaris* plants, and improved thr physio-biochemical and growth parameters of drought-stressed *P. vulgaris* plants (Figure 9).

**Figure 10:**
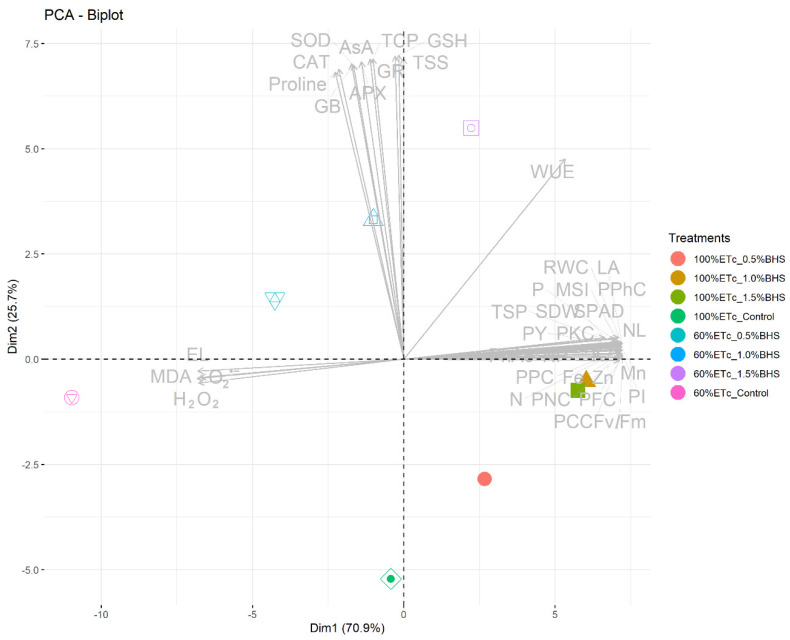
Bi-plot graph of studied parameters and treatments, showing the first two dimensions (Dim1 and Dim2) of the principal component analysis (PCA-Biplot) model in *Phaseolus vulgaris* plants treated with bee-honey solution (BHS) as foliar spray under drought stress conditions. LN: number of leaves, LA: leaf area plant, SDW: shoot dry weight, NP: number of green pods, PY: green pods yield, PPC: pod protein content, PCC: pod carbohydrates content, PFC: pod fibers content, PNC: pod N content, PPhC: pod phosphorus content, PKC: pod potassium content, PI: performance index, SPAD: Soil Plant Analysis Development (an indicator of chlorophyll content), PhAc: photochemical activity, Fv/Fm: efficiency of PSII, DW: dry weight, N: nitrogen, P: phosphorus, K: potassium, Fe: iron, Mn: manganese, Zn: zinc, FW: fresh weight, RWC: relative water content, MSI: membrane stability index, EL: electrolyte leakage, MDA: malondialdehyde (an indicator of lipid peroxidation), H_2_O_2_: hydrogen peroxide, O_2_^•−^: superoxide radical, TSS: total soluble sugars, GB: glycine betaine, AsA: ascorbate, GSH: glutathione, SOD: superoxide dismutase, CAT: catalase, GR: glutathione reductase, APX: ascorbate peroxidase, WUE: water use efficiency, and TSP: total soluble protein.

Owing to the high variation resulting from foliar spay of *P. vulgaris* plants with BHS under drought stress treatments, a biplot of principal component analysis (PCA) was conducted to show the impact of interactive treatments on all the measured traits. The two PCA-diminutions (Dim1 and Dim2) showed 70.9% and 25.7% of data variability, respectively (Figure 10).

Under the drought treatment, BHS activated the CAT, SOD, APX, and GR, and improved the levels of proline, GSH, α-tocopherol, GB, TS sugars, and AsA while EL, H_2_O_2_, MDA, and O_2_^•−^ were decreased. Moreover, the PCA-Biplot indicated that 1.5% BHS treatment achieved the highest positive influence on growth, production, and green pod quality of *P. vulgaris* plants under DtS, while 1.5% BHS or 1.0% BSH achieved the highest positive influence on growth, production, and green pod quality of *P. vulgaris* plants under normal conditions (100% of ETc) (Figure 10). Therefore, using BHS as a foliar application has a good role in enhancing the biomass production, green pod quality, and drought resistance in *P. vulgaris* plants.

## 4. Discussion

Drought stress (DtS) is more common for crop plants, especially vegetable crops grown in dry (semi-arid and arid) regions, including Egypt. It industriously limits plant performance, which includes growth and yield, as well as restricting various mechanisms of metabolic pathways [7,8,11]. Often, plants are unable to tolerate DtS by endogenously available antioxidant system components, such as the common bean used in this research which is DtS-sensitive [26,27]. Therefore, common bean plants need external support with growth stimulants, which can stimulate various processes related to the plant’s physio-biochemistry and are reflected positively in plant performance, yield quality, and plant resistance to DtS. Table 2 shows that BHS has a low pH of 4.02 and is rich in organic acids and osmotic preservers (e.g., soluble sugars and amino acids, including proline). It is also rich in essential nutrients (e.g., Se, I, Cu, Zn, Mn, Fe, S, Ca, Mg, P, and K), vitamin C (ascorbate) and B-group vitamins (B1, B2, B3, B5, B6, and B9). It also possesses a potent activity of DPPH radical scavenging (87.4%), which is utilized for assessing the capacity of antioxidants to minimize or prevent lipid peroxidation [8,20] and confer antioxidative property for BHS. In addition, [20] show that BHS contains a minimum concentration of H_2_O_2_, which has previously been reported to be efficient in promoting DtS resistance in plants [61,62]. Therefore, BHS has pivotal mechanisms and can encourage several metabolic reactions to relieve abiotic stress [8,20], including DtS in common bean. BHS is also a nutritious solution due to its phytonutrients, sugars, amino acids, and vitamins (Table 2) that support plants when they are growing under stress conditions. Thus, as a natural, inexpensive, easy-to-prepare strategy, the BHS should replace expensive synthesized applications. 

The major vital components of the BHS have previously been used individually as foliar applications with success supporting plant performance and increasing its resistance to stress, e.g., organic acids [63], soluble sugars [64,65], amino acids [66,67,68], proline [66,69], essential nutrients [70,71,72], ascorbate [73], B-group vitamins [74,75], selenium [7], and iodine [76]. In this paper, all of these components are combined into a solution (BHS) in organic forms, giving this solution a multi-mechanism for plants to adjust to stress conditions and resist stress via signaling pathways and efficient mechanisms. This multi-mechanism (e.g., organic acids, osmo-regulation, nutrition, and antioxidant systems) resulting from BHS treatment qualifies the BHS with great success as a growth multi-biostimulator utilized for common bean plants against DtS influences.

After foliar spraying, the BHS can penetrate cells from leaf surface by two pathways. The first pathway is the cuticle, and ectodesmata may function as a specific pathway [77]. The second pathway is the leaf stomata, where the BHS bioactive components can easily penetrate and pass into biosynthesizing and/or meristematic cells as bioactive leaf parts to participate in the improvement of metabolic processes, and support plants in overcoming DtS conditions [8,20].

In this paper, reducing watering from 100% to 60% of ETc hindered growth, productivity, and green pod quality of common bean plants (Figure 1 and Figure 2), deactivated the efficiency of photosynthesis machinery (Figure 3), unbalanced mineral nutrient contents (Figure 4), and disturbed leaf integrity (e.g., relative water content (RWC) and membrane stability index (MSI); Figure 5), all of which were due to stimulation of lipid peroxidation (MDA), electrolyte leakage (EL), and excess oxidants (H_2_O_2_ and O_2_^•−^) generated (Figure 5). These poor DtS-induced outcomes were associated with an increase in osmotic preservers with upregulation of various antioxidants (non-enzymatic and enzymatic) (Figure 6 and Figure 7). This induced activation of osmoregulatory substances and antioxidant capacity against oxidative damage under DtS was acted upon. Our findings confirmed those obtained in many previous reports [8,17,20].

The adverse impacts exacerbated by DtS can be attributed to the facts that (i) DtS induces osmotic stress causing cell turgor loss [8,78] and (ii) DtS stimulates excessive ROS production causing oxidative stress [8,79]. However, leafy application of BHS (especially at 1.0% (BHS-2) for normal plants and 1.5% (BHS-3) for stressed plants) attenuated the adverse impacts of DtS on growth, productivity, and green pod quality of common bean, thereby improving these traits, all of which outperformed those of well-watered plants without BHS, which increased WUE. WUE was noticeably higher with BHS-3 under DtS than with BHS-2 under normal conditions (Figure 1). Restoration of growth, production, and green pod quality of common bean plants grown under DtS by BHS application displayed that this multi-biostimulator has multi-mechanism (e.g., osmoprotective compounds, antioxidants, including B-group vitamins and ascorbate, as well as mineral nutrients, including Se and I), supporting plants to restore their growth and development due to DtS attenuation. Our findings confirmed those obtained in many previous reports [8,20].

In this study, leaf content of chlorophyll (SPAD value) and efficiency of photosynthesis (photochemical activity (PhAc), F_v_/F_m_, and performance index (PI)) were reduced under irrigation at 60% of ETc (DtS) (Figure 3). This indicates that the excess ROS induced by DtS (Figure 5) stimulated chloroplast chlorolysis and chlorophyll degradation, as well as PSII photoinhibition in common bean plants. Our findings confirm those of [8,80,81]. However, SPAD value (chlorophyll content), PhAc, PI, and F_v_/F_m_ were restored in stressed *P. vulgaris* plants by foliar spray with BHS [8,20]. These outcomes were linked, in this study, to mineral nutrient recovery, maintenance of integrity of cell membranes, and the restoration of leaf RWC by BHS supplementation (Figure 4 and Figure 5). Possibly, BHS attenuated the adverse impacts, and common bean plants responded efficiently to DtS through some BHS mechanisms, including mineral nutrient recovery (Figure 4), as well as upregulation of osmoregulatory compounds (Figure 6) and various antioxidants (non-enzymatic and enzymatic) (Figure 6 and Figure 7) to scavenge excess ROS (O_2_^•−^ and H_2_O_2_), thereby minimizing lipid peroxidation (MDA) and EL. These effective BHS mechanisms come on the grounds that BHS contains different nutrients (Table 2) for the maintenance of intercellular ion hemostasis needed for biosynthesis of chlorophyll in common bean leaves to activate photosynthesis. Another set of mechanisms, BHS osmoregulatory compounds, can contribute to RWC for healthy metabolic processes, as well as enabling BHS vitamins and antioxidant capacity (89.4%) to support the common bean plant antioxidant defense system (Figure 6 and Figure 7). In addition, the increased photosynthetic efficiency was linked, in this study, to increased proline content (Figure 3 and Figure 6). As reported in [66,82], proline is a greater cumulative substance in plants that contributes to increased photosynthetic efficiency and adenosine triphosphate (ATP) generation. In this regard, the administration of BHS further raised proline content during DtS. Therefore, the enhanced proline and soluble sugars with BHS fortification, in this work, under the conditions of DtS might corroborate their relevance as osmoregulators that impart resistance in the plant to DtS. 

In this study, due to DtS (irrigation at 60% of ETc), impaired nutrient availability (decreased nutrient contents; Figure 4) leads to nutrient deficiencies such as those seen on common bean plants (data not shown). This undesirable impact is attributed to osmotic and oxidative stresses, which disturb nutrient availability, absorption, transport, and metabolism [8,9], reflecting chlorophyll degradation and nutrient deficiency symptoms. However, BHS foliar supplementation stimulated ionic balance and boosted plant contents of nutrients under stress (Figure 4). This could be due to increased root absorption surfaces as a result of the boosted volume of the root system (data not shown), and/or reinforced accumulation of osmotic preservers (Figure 6) for balancing the osmotic pressure in cell organelles. Therefore, cell turgor is maintained and the absorption of different nutrients is promoted [8,20,83] in favor of RWC.

As physiological measures, RWC is an indicator of available water content in metabolizing tissues [84], while MSI and EL are indicators of membrane integrity status [8,85]. The stressed leaf tissue recovery (i.e., increased RWC, cell turgor and MSI, membrane integrity with reduced EL) was mediated by BHS (Figure 5). Due to the application of BHS, RWC improvement in stressed common bean plant tissues and cells preserved cell turgor by accumulating more osmotic preservers like glycine betaine, proline, and soluble sugars (Figure 6) and/or altered cell wall elasticity [9,86]. This authorized continued metabolic activities as powerful mechanisms of resistance to DtS in common bean plants. RWC promotion by foliar spray with BHS is closely associated with WUE promotion in common bean plants (Figure 1). In this investigation, the osmotic preservers and various antioxidants (enzymatic and non-enzymatic) (Figure 6 and Figure 7) were increased by foliar supplementation of BHS to protect the plasma membrane from lipid peroxidation (MDA) and EL by decreasing the oxidant (H_2_O_2_ and O_2_^•−^) contents (Figure 5). These findings were associated with increased MSI, decreased EL and photooxidation, and enhanced membrane integrity against oxidative damage [8,20], thus improving components of the common bean plant growth, production, and green pod quality under DtS (Figure 1).

In this study, the plant defense mechanisms (viz, the antioxidant defense system; ADS), including the synthesis of osmotic preservers (soluble protein, glycine betaine, soluble sugars, and proline; Figure 6) and activities of non-enzymatic and enzymatic antioxidants (AsA, GSH, αToc, SOD, CAT, GR, and APX; Figure 6 and Figure 7), were ameliorated in BHS-treated plants under normal conditions or under DtS. ADS protects common bean plants from DtS damage via osmotic adjustment (upregulation of osmoprotectants) and excess ROS scavenging [8,20]. This upregulation and increase of osmotic-protective substances probably led to the absorption and then dissociation of BHS as a multi-biostimulator with high contents of osmotic-protective substances (Table 2). Our study outcomes exhibited that DtS markedly raised the ADS components (AsA, GSH, αToc, SOD, CAT, GR, APX, and osmoprotectants) to enable common bean plants to partially tolerate DtS effects. However, foliar application of BHS on plants further increased the components of this ADS, enabling the plants to tolerate oxidative damage as confirmed by suppressed MDA, EL, H_2_O_2_, and O_2_^•−^ levels (Figure 5), and conferring full resistance to DtS. Due to their antioxidative properties, GSH and AsA have a notable role in protective defenses under stresses, including DtS, markedly minimizing oxidative damage lipid peroxidation [7]. Owing to its propensity to contribute electrons in several enzymatic/non-enzymatic reactions, AsA is an exceptionally effective ROS scavenger. As reported in [87], by scavenging O_2_^•−^ and OH^−^ directly, cell membranes are protected under stress by AsA. In this study, higher GSH and AsA levels under stress contributed to BHS-induced reductions in H_2_O_2_ and MDA levels. Therefore, the equilibrium of the GSH and AsA pools must be regulated rigorously with sufficient activity of APX, which was also enhanced in this investigation by BHS (Figure 6 and Figure 7) to boost the cell antioxidant capability and prevent oxidative stress-induced damage [88]. Growing levels of GSH and AsA as a result of BHS administration suggest a perfection in the “AsA-GSH cycle”, which combats excessive ROS formation. This cycle regulates the amount of cell H_2_O_2_. Associating with MDHAR and DHAR, GR initially offers substrates for APX by generating GSH and AsA [7]. Reportedly, αToc, as a nonenzymatic lipophilic antioxidant, can scavenge several ROS under stress [82]. The greater content of αToc achieved by BHS administration (Figure 6) was accompanied by decreased oxidative stress indicators (O_2_^•−^ and H_2_O_2_) and MDA levels, which represent the plasma membrane integrity. Reported also, plasma membrane phospho-lipids are a specific target of several oxidants, but αToc saliently repairs membranes by suppressing lipid peroxidation through decreasing the development of oxidized phospholipids that may hypothetically interfere with membrane fusion processes. These antioxidants provide resistance to DtS that stimulate ROS generation at different development stages [89]. BHS can minimize damage to cell membranes by strengthening the photosynthesis machinery, increasing ROS quenching, enhancing morpho-physiological indicators, and upregulating the enzymatic defense system as shown in this study (Figure 1, Figure 2, Figure 3, Figure 4, Figure 5, Figure 6 and Figure 7). Therefore, BHS, as a multi-biostimulator that has multi-mechanism for stressed plants, can enhance development and productivity in plants and has affirmative implications for farming productivity features.

In association with non-enzymatic systems in the defense of plants under DtS, the enzymatic system plays an irreplaceable role. Among enzymes, SOD protects cell components from the mischievous impacts of O_2_^•−^ by converting it into O_2_ and H_2_O_2_. Thus, the danger of OH• production by means of a metal-catalyzed Habere–Weiss type reaction is diminished [90]. As reported in [8,91], an increased activity in antioxidant machinery of Se- or BHS-fortified plants has been explored under DtS conditions. Associating with GSH-Px and SOD, additional enzymes, including APX, CAT, GR, etc., can operate when *P. vulgaris* plants were fortified with BHS to reduce damage from DtS. In this study, these enzymes acted primarily to remove O_2_^•−^ and H_2_O_2_ (Figure 5 and Figure 7). APX serves as a signaling molecule for the downregulation of ROS, while excess ROS can be eliminated by CAT (mostly located in peroxisomes) [92]. BHS was discovered, in this investigation, to strongly reactivate all enzyme activities due to the increased levels of antioxidative metabolism and enzyme activity in chloroplasts. This was consistent with BHS’s capacity to minimize the levels of H_2_O_2_ and MDA in chloroplasts. These findings demonstrated, under DtS, that Se attenuates membrane damage in chloroplasts by enhancing the scavenging capacity of ROS, hence keeping PSII protected from oxidative stress [8,20].

Finally, the adverse influences of DtS may exceed the resistance naturally present in stressed common bean plants since components of plant ADS do not meet adequate defense requirements against stress. However, in this study, the osmoprotectants, antioxidants, nutrients, and vitamins present in BHS reinforced the efficiency of ADS. The enhancement of ADS enabled plants to perform efficiently under stress [8,20] due to improvements in physiological processes, and thus biochemical attributes, leading to increased resistance in common bean plants to DtS.

## 5. Conclusions

Differences in physiological-biochemical and metabolic responses were explored, in this study, between bee-honey solution (BHS)-fortified and non-fortified common bean plants. Exogenously sprayed BHS (at 1.0% for normal plants and at 1.5% for stressed plants) maximized levels of various antioxidants (non-enzymatic and enzymatic) and osmotic protectants, all minimized oxidants (hydrogen peroxide and superoxide) and oxidant damage in terms of minimized malondialdehyde and electrolyte leakage, and improved nutritional balance, tissue integrity, and photosynthetic efficiency, resulting in increased growth, productivity, and yield quality of *Phaseolus vulgaris* plants under drought stress. Due to its richness in antioxidants, osmoprotectants, nutrients, and vitamins, BHS was explored to be an efficient ecofriendly strategy to attenuate drought stress (60% of ETc) damages for sustainable common bean production and yield quality in semi-arid and arid regions. More studies are required on the application of honey in the agricultural sector to explore the precise mechanisms that make the BHS-treated plants become highly efficient in resisting abiotic stresses.

## Figures and Tables

**Table 1:** Some initial physicochemical properties of soil in the upper 0–30 cm layer.

Particle Size Distribution				Bulk Density (g cm^−3^)	K_sat_ cm h^−1^	FC (%)	WP (%)	AW (%)	pH	ECe (dS m^−1^)	OM (%)	CaCO_3_ (%)
Sand %	Silt %	Clay %	TC									
20.2	38.4	41.4	CL	1.38	1.18	34.0	19.8	15.8	7.726	2.54	1.32	3.38

TC = Texture class, CL = Clay loam, FC = Field capacity, WP = wilting point, AW = Available water, OM = Organic matter, and K_sat_ = Hydraulic conductivity.

**Table 2:** Chemical analysis of fresh raw clover honey used in this study.

Property/Component	Unit	Value
Moisture	%	17.2 ± 0.65
Organic acids		0.51 ± 0.02
pH		4.02 ± 0.14
Osmoprotectants:		
Proline	mg kg^−1^ FW	47.8 ± 1.89
Total soluble sugars	%	81.9 ± 2.42
Amino acids		0.34 ± 0.01
Mineral nutrients:		
Potassium (K)	mg kg^−1^ FW	460 ± 11.2
Phosphorus (P)		49.8 ± 1.85
Magnesium (Mg)		83.6 ± 2.61
Calcium (Ca)		70.2 ± 1.92
Sulphur (S)		75.5 ± 1.99
Iron (Fe)		69.5 ± 1.84
Manganese (Mn)		8.62 ± 0.30
Zinc (Zn)		5.64 ± 0.18
Copper (Cu)		4.58 ± 0.15
Iodine (I)		80.8 ± 2.44
Selenium (Se)		0.94 ± 0.04
Antioxidants and Vitamins:		
Ascorbic acid (vitamin C)	mg kg^−1^ FW	25.0 ± 0.55
Thiamine (B1)		0.15 ± 0.00
Riboflavin (B2)		0.19 ± 0.00
Niacin (B3)		1.70 ± 0.07
Pantothenic acid (B5)		1.05 ± 0.05
Pyridoxine (B6)		2.21 ± 0.11
Folate (B9)		0.22 ± 0.01
DPPH radical-scavenging activity	%	87.4 ± 2.58

## Data Availability

The data presented in this study are available upon request from the corresponding author.
